# Snapshot Imaging Spectrometer Based on Pixel-Level Filter Array (PFA)

**DOI:** 10.3390/s21072289

**Published:** 2021-03-25

**Authors:** Yunqiang Xie, Chunyu Liu, Shuai Liu, Weiyang Song, Xinghao Fan

**Affiliations:** 1 Changchun Institute of Optics, Fine Mechanics and Physics, Chinese Academy of Sciences, Changchun 130033, China; xieyunqiang131@163.com (Y.X.); liushuai@ciomp.ac.cn (S.L.); swy_ucas@163.com (W.S.); ccfanxh@163.com (X.F.); 2 University of Chinese Academy of Sciences, Beijing 100049, China; 3 Key Laboratory of Space-Based Dynamic & Rapid Optical Imaging Technology, Chinese Academy of Sciences, Changchun 130033, China

**Keywords:** snapshot spectral imaging, pixel-level filter array, simple implementation, low computational burden

## Abstract

Snapshot spectral imaging technology plays an important role in many fields. However, most existing snapshot imaging spectrometers have the shortcomings of a large volume or heavy computational burden. In this paper, we present a novel snapshot imaging spectrometer based on the pixel-level filter array (PFA), which can simultaneously obtain both spectral and spatial information. The system is composed of a fore-optics, a PFA, a relay lens, and a monochromatic sensor. The incoming light first forms an intermediate image on the PFA through the fore-optics. Then, the relay lens reimages the spectral images on the PFA onto the monochromatic sensor. Through the use of the PFA, we can capture a three-dimensional (spatial coordinates and wavelength) datacube in a single exposure. Compared with existing technologies, our system possesses the advantages of a simple implementation, low cost, compact structure, and high energy efficiency by removing stacked dispersive or interferometric elements. Moreover, the characteristic of the direct imaging mode ensures the low computational burden of the system, thus shortening the imaging time. The principle and design of the system are described in detail. An experimental prototype is built and field experiments are carried out to verify the feasibility of the proposed scheme.

## 1. Introduction

Imaging spectrometers are dedicated devices used for capturing both spatial and spectral information simultaneously. The captured information forms a three-dimensional (3D) datacube denoted by two-dimensional space and one-dimensional spectrum. Compared with general optical imaging, the additional spectral information can be applied to many fields, such as mineral exploration, identification of artwork, medical diagnosis, and agricultural detection [1,2,3].

In view of the inherent limitation of two-dimensional (2D) imaging sensors in capturing 3D spectral images, most conventional imaging spectrometers are scanning-based systems, which either scan in the spatial domain or the spectral domain in a time-sequential fashion, and thus need multiple exposures to capture full spectral images [4,5,6,7]. When acquiring dynamic targets requiring a high temporal resolution, this can be restricted by the scanning speed of the spatial-scanning imaging spectrometers, leading to severe motion artifacts and pixel misregistration problems. Another drawback of the spatial-scanning imaging spectrometer is the short pixel dwell time, which limits the amount of light received per pixel, and thus reduces the signal-to-noise ratio (SNR) of the system. Spectral-scanning imaging spectrometers based on filter wheel or variable filters, such as acousto-optic filters (AOTF) [8] and liquid crystal tunable filters (LCTF), can provide a high data acquisition speed, but suffer from low transmission efficiency [9].

With the recent advancement of large-format 2D focal plane arrays (FPA), various snapshot imaging spectrometers have been developed. Snapshot imaging spectrometers can acquire spatial and spectral data within the single integration time of the imaging sensor [10,11,12]. These spectral imagers have more advantages than existing scanning spectrometers because of their longer pixel dwell time, ensuring their application in low-light levels and high-speed imaging. Additionally, compared with scanning systems, snapshot imaging spectrometers can mitigate motion artifacts and simplify image mosaicking. The capability of capturing data during a single integration time of the imaging sensor guarantees a great extent of synchronization between multiple snapshot imaging spectrometers, a property utilized in remote sensing to acquire estimates of bidirectional reflectance-distribution function by a fleet of spatially, directionally, and temporary coordinated devices [13].

Snapshot imaging spectrometers can be mainly classified into two categories—direct imaging and computational imaging [14,15,16]. The most typical direct imaging systems include integration-field spectroscopy (IFS) [17,18], the multispectral sagnac interferometer (MSI) [19], the image mapping spectrometer (IMS) [20,21], and the image replicating imaging spectrometer (IRIS) [22,23]. These systems have the advantage of possessing simple data reconstruction algorithms, which need less computational loads and thus can help to display and analyze spatial and spectral information at high frame rates [24]. However, most of the direct imaging systems apply stacked dispersive or interferometric elements to split spectral channels, making the system cumbersome. In comparison, computational imaging spectrometers first acquire a batch of coded projections of the 3D data, from which the spectral images can be reconstructed later. Typical computational imaging spectrometers include computed tomographic imaging spectrometry (CTIS) [25], coded aperture snapshot spectral imagers (CASSI) [26,27], and snapshot hyperspectral imaging Fourier transform (SHIFT) [28]. Computational imaging systems are usually compact and have more spectral bands than direct imaging systems. However, because of the massive computational requirements to reconstruct spectral images [29,30,31], computational imaging systems cannot display the full-resolution datacube in real time, thus preventing their application in time-crucial projects. Thus, a snapshot imaging spectrometer meeting the conditions of simple implementation, low computational complexity, and high reconstruction performance is of great research value.

In this paper, we present a novel snapshot imaging spectrometer that can dynamically capture both spatial and spectral information. The core idea of our system is based on a pixel-level filter array (PFA), which relies on lithography and vacuum multilayer film technologies to realize the accuracy required to shrink the size of each filter down to that of a single pixel of imaging sensor. A group of a certain number of filters forms the filter cell, which is repeatedly copied to cover the entire substrate. We finally chose the re-imaging scheme after a comprehensive comparison with the once-imaging scheme. Our system is principally composed of a fore-optics, a relay lens, a PFA, and a monochromatic sensor. One to one mapping correspondence will be established between filters on the PFA and pixels on the monochromatic sensor in the stage of alignment. The spectral images of the 3D datacube captured by the system can be easily extracted from the raw image by a simple remapping algorithm based on the correspondence. Compared with the existing snapshot imaging spectrometers, as the data are directly obtained by our system, the post-processing requires less of a computation load, enabling its application in time-crucial tasks. Through the use of PFA, our system removes stacked dispersive or interferometric elements, making it advantageous over the other snapshot imaging spectrometers because of the qualities of compactness, light weight, and low cost. In addition, by getting rid of grating and prism, the system possesses a high energy efficiency. The datacube acquired by our system is 160 × 128 × 16 (x, y, λ). The spectrum ranges from 900 nm to 1700 nm (short-wave infrared), and is divided into 16 spectral channels with an average sampling interval of 30 nm. It should be noted that in addition to the similar uses for visible light detection, short-wave infrared has the ability to observe through clouds and fog. We further built an experimental prototype based on these basic principles and our optical design. A field test was also carried out to evaluate the feasibility of the presented system.

## 2. Pixel-Level Filter Array (PFA)

**Figure 1:**
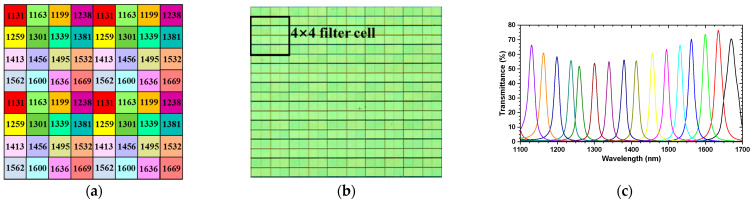
(**a**) Geometric pattern of the 16-channel pixel-level filter array (PFA) we selected, (**b**) microscope image of the 16-channel PFA, and (**c**) transmittance curves of the PFA.

A PFA is composed of a large number of periodically repeated filter cells that are deposited on a substrate. The substrate can be optical glass or polymer film. Each filter cell is composed of multiple micro filters that are the same size as a pixel of the imaging sensor. Each micro filter only allows a narrow band light centered at a specific wavelength to pass through. In this paper, we selected a PFA containing 160 × 128 filter cells. Each filter cell was composed of 16 micro optical filters (4 × 4), 15 μm × 15 μm in size. In order to match the PFA, we chose a monochromatic sensor (GHOPTO; GH-SWGnet-15) with 640 × 512 pixels, 15 μm × 15 μm in size, to capture data. The PFA allowrf light of wavelengths centered at 1131, 1163, 1199, 1238, 1259, 1301, 1339, 1381, 1413, 1456, 1495, 1532, 1562, 1600, 1636, and 1669 nm with a bandwidth of 25 nm to pass through. Figure 1a shows the geometric pattern of the 16-channel PFA we selected, and the figure in each square represents the central wavelength of the light passing through the corresponding filter. Figure 1b,c shows the microscope image of the PFA and its measured response to light, respectively.

## 3. Optical System Design and Optimization

### 3.1. Selection of Optical Scheme

According to the characteristics of PFA, once-imaging and re-imaging schemes were considered separately.

**Figure 2:**
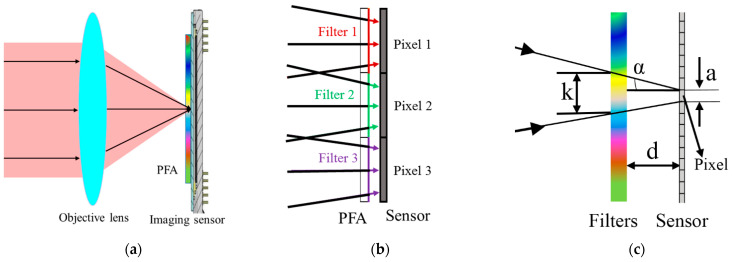
Once-imaging scheme: (**a**) Optical structure, (**b**) the correspondence between filters and pixels, and the (**c**) generation of crosstalk.

For the once-imaging scheme, the system was composed of an objective lens, a PFA, and an imaging sensor. As shown in Figure 2a, the PFA was placed in proximity to the imaging sensor, with the coated-side towards the pixels. When working, the incoming light beams from the object were imaged by the objective lens, spectrally mixed by the PFA, and finally captured by the imaging sensor. Figure 2b shows the corresponding relationship between the filters and pixels. This scheme possesses the advantage of a simple implementation and compact structure. However, there is an obvious flaw in the system. When assembling the PFA and imaging sensor, in order to protect the imaging sensor and the filters on the PFA to prolong the service life, the PFA could not be attached to the pixels, which means that there needed to be a gap between them. According to the gap between the protective glass and pixels of existing detector, this was about 0.5 mm. Because of the gap, severe crosstalk between different optical channels occurred. Figure 2c is a schematic diagram of the generation process of crosstalk, where k is the diameter of the facula on the PFA projected by the light converging on a pixel of imaging sensor, a is the diameter of a pixel, d is the gap between the filters on the PFA and imaging sensor, and α is the aperture angle of the objective lens in the image space. Based on the geometrical relation, we can get the following conclusion:

(1) k≈a+2d tanα,

According to the PFA and imaging sensor we adopted, and the objective lens commonly used, we set a to 15 μm, d to 0.5 mm, and α to 7°. Calculated by Equation (1), we find that k is about 187 μm, which is the size of 12 filters. Based on our calculation, one pixel will receive light passing through at least 12 filters, causing severe spectra crosstalk, so the once-imaging scheme is not desirable.

**Figure 3:**
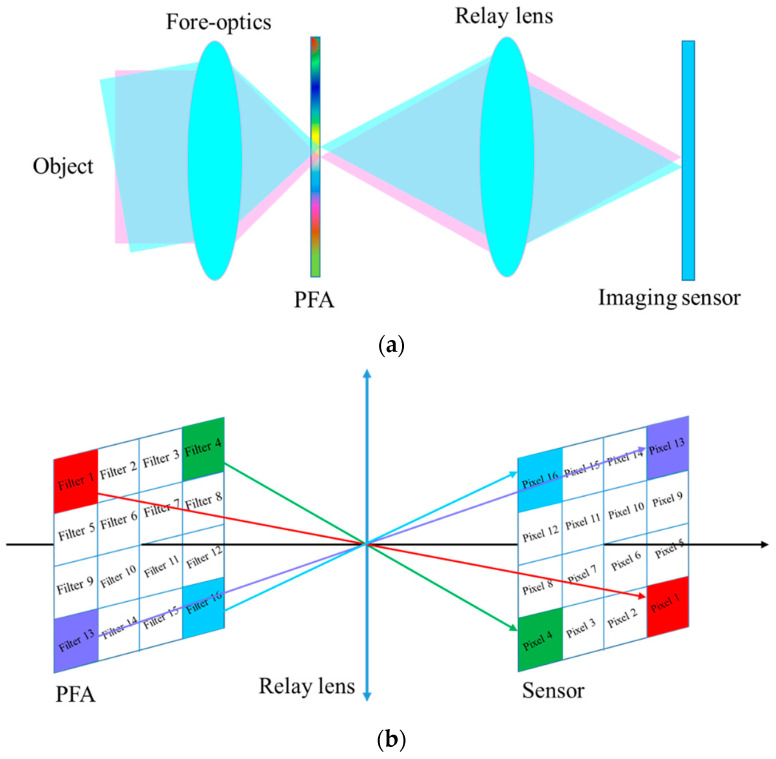
(**a**) Optical structure of re-imaging scheme and (**b**) one to one mapping correspondence between the filters on the PFA and pixels on imaging sensor.

In order to solve the problem of crosstalk between different optical channels, a re-imaging scheme is proposed in this paper. In this scheme, a high-precision relay lens was introduced into the optical path, as shown in Figure 3a. The system consisted of a fore-optics made of an objective lens, a PFA, a relay lens, and an imaging sensor. In the process of operating, the incoming light beams from the object first formed an intermediate image on the coated-side of the PFA by the fore-optics. Based on the central wavelength and bandwidth, the PFA then optionally transmitted through the spectrum of the intermediate image, which was then reimaged on an imaging sensor by a relay lens. Each filter on the PFA corresponded to a pixel of the imaging sensor, and the relationship between them was an optical conjugation of the relay lens, as shown in Figure 3b. Before spectral imaging, spectral calibration was implemented to assign the pixels on the sensor to a unique wavelength, and to ensure the same periodicity of the pixel center wavelength as the PFA. Then, the 3D datacube could be acquired in a single exposure time by the imaging sensor. The spectral image at each band was finally recovered directly by extracting the corresponding pixels periodically from the raw image using a simple algorithm. No complex iterative reconstruction algorithm was required. For example, the spectral image of the first band could be obtained with the following equation:

(2) $$I_{1}\left(x,y\right)=R\left(1+4\left(x-1\right),1+4\left(y-1\right)\right)$$

where *R* is the raw image, x∈[1,160], y∈[1,128].

**Figure 4:**
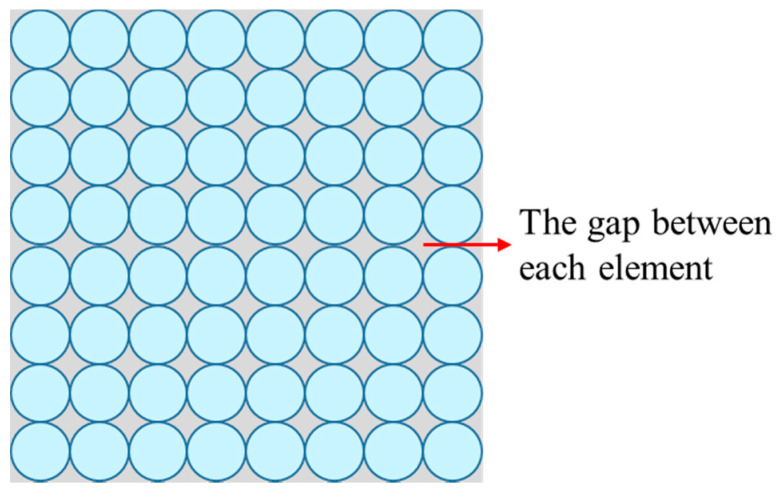
Schematic diagram of the gap between each element in microlens array, digital micromirror, and optical fiber bundle.

To ensure the corresponding relationship between the filters and pixels, the relay lens needed to possess a fine image quality and minimal distortion. In addition, as the filter cells of the PFA and pixels of the imaging sensor we choose were the same size, the lateral magnification of the relay lens was equal to −1. This scheme enabled the system to avoid the crosstalk caused by the gap between the PFA and imaging sensor. In addition, as existing snapshot imaging spectrometers rely on microlens array, digital micromirror, or an optical fiber bundle to multiplex the light spatially, energy loss during the imaging process occurring as a result of the gap between each element is shown in Figure 4. In comparison, our system enjoyed a high energy efficiency through the use of PFA.

### 3.2. Fore-Optics

**Figure 5:**
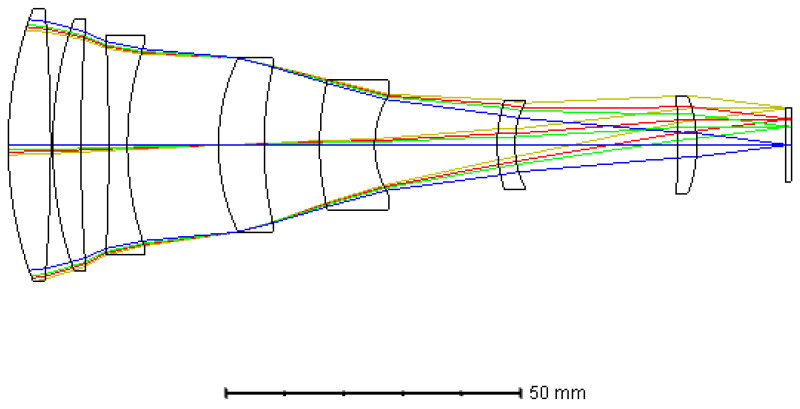
Optical layout of the fore-optics.

The fore-optics was used for collecting the scene to form the intermediate image, and it was not necessary to take into account the characteristics of the PFA too much. It projected an image of the scene on the PFA, and from there, the relay lens reimaged the intermediate image to the monochromatic sensor. The specifications of the fore-optics are shown in Table 1. As the micro optical filters on the PFA had the potential for a spectral shift, the main light in each field of view had to be vertically incident on the PFA. Thus, the fore-optics should be designed in the form of a telecentric path in the image space to work properly with the PFA. For this purpose, we set the last lens of the system as a positive lens to control the angle of the light relative to the optical axis. Because of the wide working waveband, the fore-optics was originally designed with a combination of three types of glasses (H-ZPK1A, H-LAK52, and H-ZF88) in order to eliminate partial dispersion and minimize residual chromatic aberrations based on Equations (2) and (3):

(3) $$\sum_{i=1}^{n}h_{i}^{2}\left(\varphi_{i}/v_{i}\right)=0,$$

(4) $$\sum_{i=1}^{n}h_{i}^{2}\left(\varphi_{i}/v_{i}\right)P_{i}=0,$$

where $h_{i}$ is the ray height of the ith lens, $P_{i}$ is the relative partial dispersion of ith lens, and $\varphi_{i}$ and $v_{i}$ are the power and dispersion coefficient of the ith lens, respectively. We then optimized the system using ZEMAX and the final optical layout is shown in Figure 5. The fore-optics is composed of seven lenses with the coating surface of PFA at the image plane. As is shown in Figure 5, this optical system was designed around a common optical axis so as to minimize the sensitivity of the assembly and alignment. The distances between the different lenses were controlled to ensure adequate mechanical clearances for the spacers and to balance the coma and astigmatism of the optical system. All of the surfaces of the seven lenses were spherical, which has the advantage of simple processing and low cost. The total axial length of this optical layout was about 133 mm.

**Figure 6:**
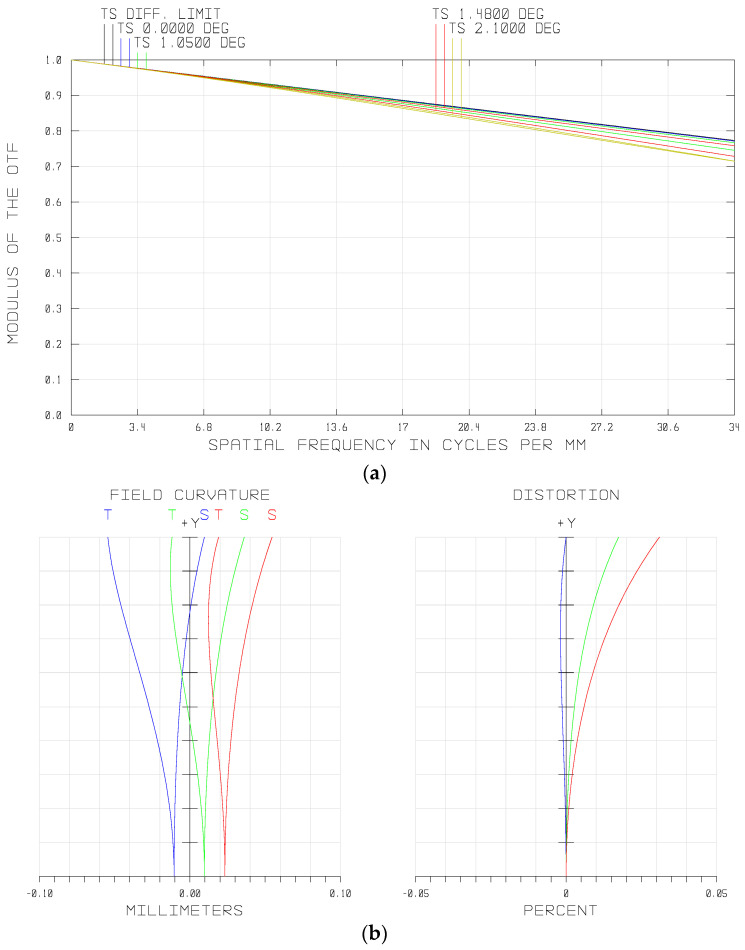

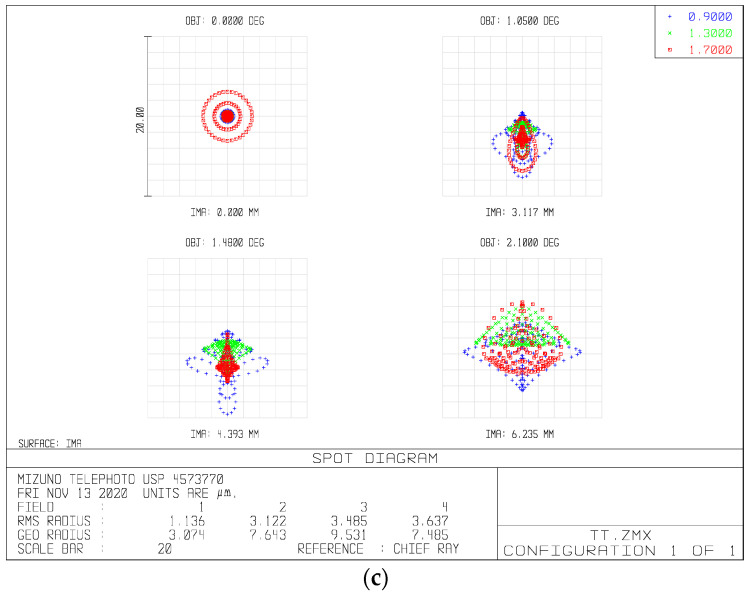
Optical performances of the fore-optics: (**a**) modulation transform function (MTF) of the system, (**b**) spot diagram, and (**c**) field curvatures and distortions of the different wavelengths.

The modulation transform function (MTF) represents the image quality of the imaging system. The MTF curve of the fore-optics in the waveband of 900–1700 nm is shown in Figure 6a. It can be seen that the MTF at the full field of view (FOV) exceeded 0.70 at the Nyquist frequency of the PFA, i.e., 34 lp/mm, approaching the diffraction limitation. Figure 6b shows the spot diagram of the whole semi-FOV of the system, from which we can see that the maximum root mean square (RMS) diameter was less than one single pixel size (15 μm) over the full FOV.

Optical distortion is the kind of aberration that deforms the image plane. By adjusting the shape and magnification factors of the lenses in this system, we restricted the optical distortion at a low level. As shown in Figure 6c (right), optical distortion of the system was controlled within a range of less than 0.05%. Furthermore, the maximum value of the field curvature was less than 0.1 mm. Overall, all aberrations of the fore-optics were well corrected.

### 3.3. Relay Lens

**Figure 7:**
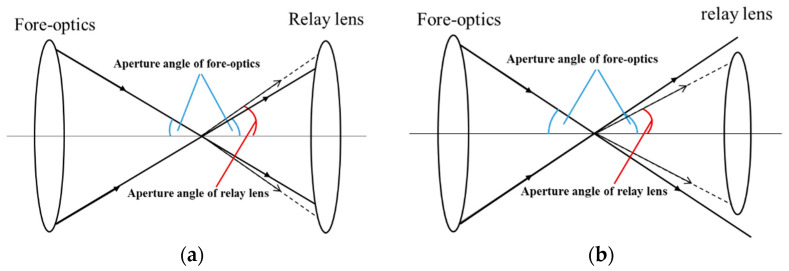
Aperture angle match diagram. (**a**) The aperture angle of the relay lens is greater than that of the fore-optics, and no vignetting will occur. (**b**) The aperture angle of the relay lens is less than that of the fore-optics, causing vignetting.

In order to avoid vignetting, which will reduce the signal-to-noise ratio (SNR) of the system when combining the relay lens and fore-optics into an overall system, the aperture angle of the relay lens in the object space cannot be less than that of the fore-optics in the image space. The schematic diagram of the aperture matching is shown in Figure 7, where solid lines represent the output light of the fore-optics with the maximum aperture angle and the dotted lines represent the incident light of the relay lens with the maximum aperture angle. As the image space aperture angle of the fore-optics we designed was 7°, we set the aperture angle of the relay lens in the object space as 9° in order to have an abundant allowance.

**Figure 8:**
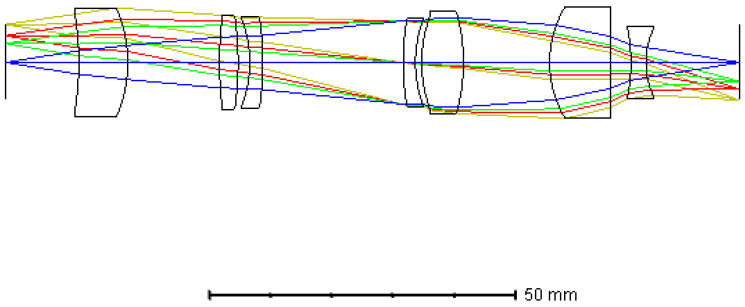
Optical layout of the relay lens.

As the shape and size of the filters on the PFA were the same as the pixels of the imaging sensor selected, and there was a one-to-one mapping between them, the lateral magnification of the relay lens was required to be −1, and the optical distortion should be strictly controlled. Otherwise, the corresponding relationship would be disordered, resulting in crosstalk between the adjacent spectral channels of the PFA. To ensure that the magnification of the relay lens was strictly equal to −1 in each field, the system was optimized by the multi-configuration function of ZEMAX, and only the FOV was different among each configuration. We set 0.3 times, 0.5 times, 0.7 times, and full FOV in the different configurations, separately, during optimization. The image height of each configuration was set as the opposite of the object height of the corresponding field. The system was designed as a quasi-symmetric structure in order to reduce distortion. In order to combine it with the fore-optics and avoid the spectral shift of the filters on PFA, the relay lens was designed in the form of a telecentric path in the object space. The glass combination of H-ZF12, H-ZLAF68C, and H-ZPK1A was initially selected to reduce the chromatic aberration and secondary spectrum according to the theory in Section 3.2. We optimized the system using ZEMAX. The total axial length of the relay lens was about 120 mm. Figure 8 below shows the final optical layout of the relay lens.

**Figure 9:**
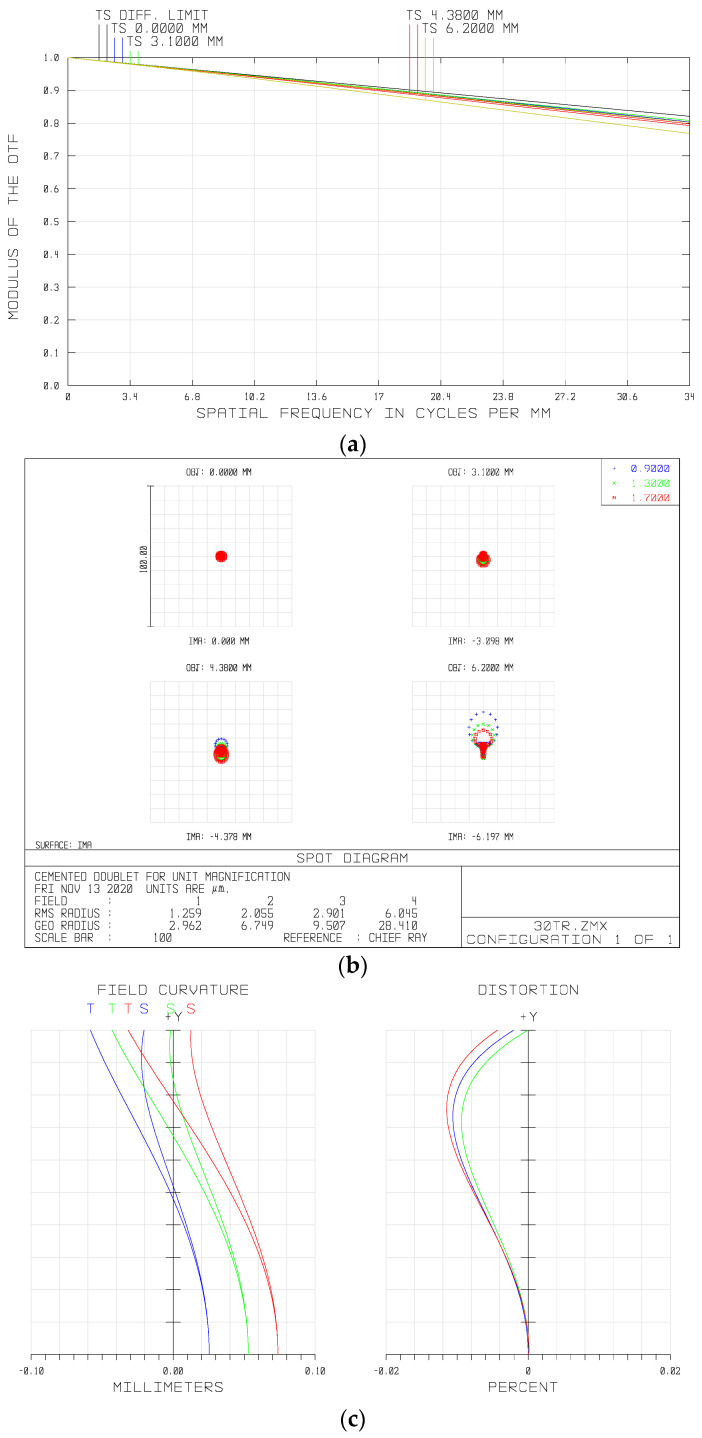
Optical performances of the relay lens: (**a**) MTF of the system, (**b**) spot diagram, and (**c**) field curvatures and distortions of different wavelengths.

The MTF and spot diagram of the relay lens are shown in Figure 9. It can be seen that the MTF of each FOV was greater than 0.76 at the Nyquist frequency of the imaging sensor, i.e., 34 lp/mm, approaching the diffraction limitation. The maximum root mean square (RMS) diameter was less than one single pixel size (15 μm) over a full FOV.

The distortion of the optical system would lead to the deformation of the real image relative to the object. Therefore, if the distortion of the relay lens was too large, the one-to-one mapping between the filters and detector pixels would be disordered, leading to crosstalk at the edge of the image plane. The distortion of the relay lens is shown in Figure 9c. It can be seen that the relative distortion over the full FOV of the relay system was less than 0.012%. The relative distortion of optical system is given by

(5) $$\mathrm{distortion}=\frac{y^{\prime }-y}{y}\cdot 100\%,$$

where $y^{\prime }$ is the real image height and y is the ideal image height. Calculated by the formula of relative distortion, the maximum deformation of the real image relative to the ideal image height, i.e., the object height in this system was 0.744 μm, which is less than 1/20 of one single pixel size. This means that the distortion of the relay lens would cause little crosstalk.

### 3.4. Overall Optical System

**Figure 10:**
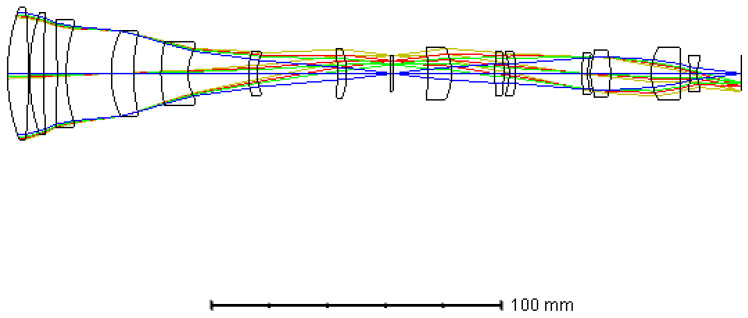
Optical layout of the overall system.

**Figure 11:**
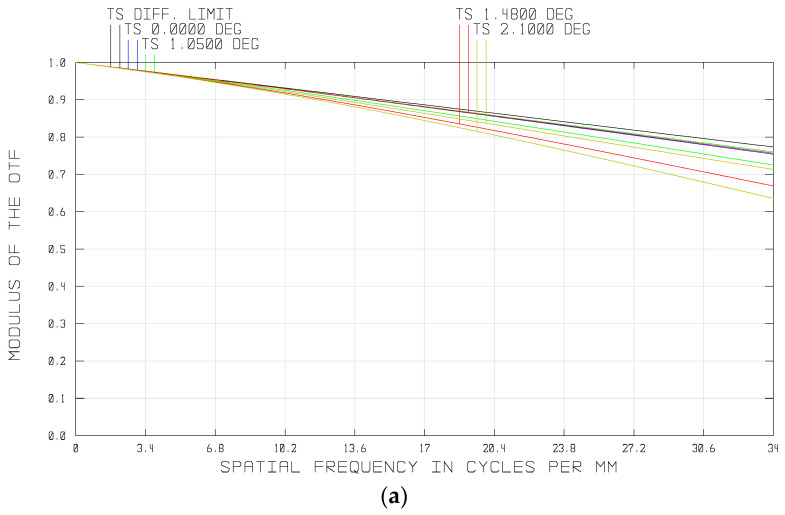

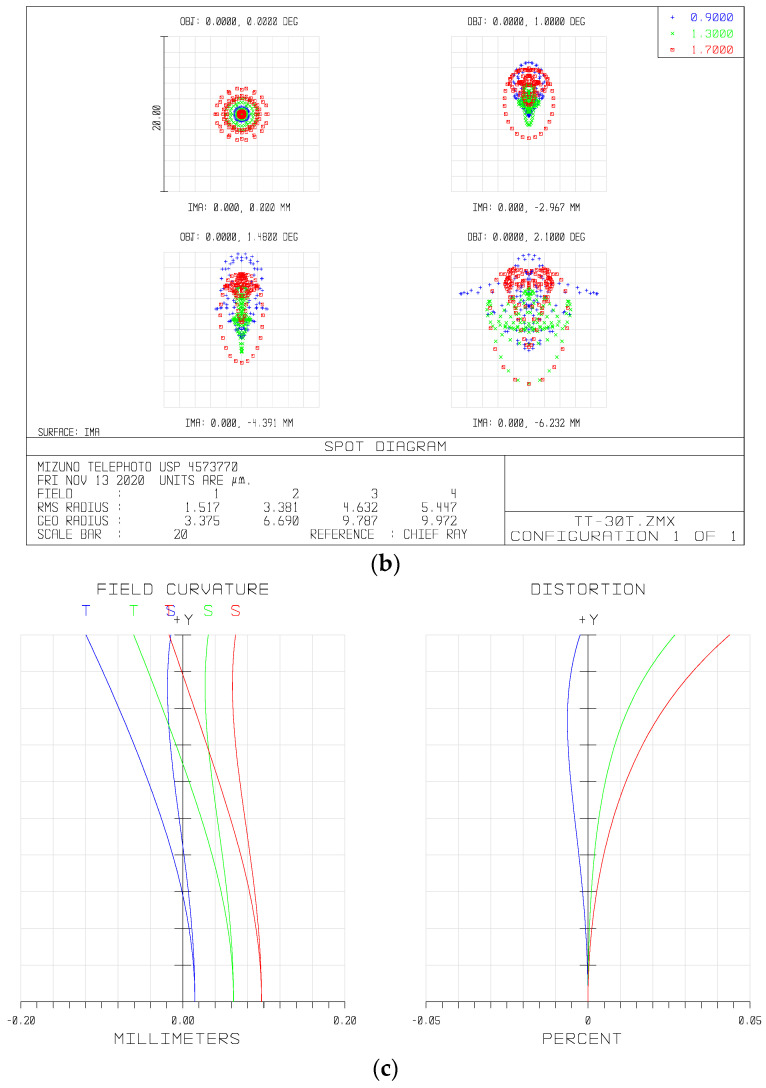
Optical performances of the overall system: (**a**) MTF of the system, (**b**) spot diagram, and (**c**) field curvatures and distortions of the different wavelengths.

We combined the fore-optics and the relay lens into an overall system, and the optical layout is shown in Figure 10. The total axial length of the overall optical system was about 253 mm. Figure 11 shows the optical performances of the system. As shown in Figure 11a, the MTF of the overall system was greater than 0.6 at the Nyquist frequency of the imaging sensor, approaching the diffraction limitation. Figure 11b is the spot diagram of the whole semi-FOV, which shows that the maximum diameter is less than one single pixel size (15 μm). The optical distortion of the system was also well corrected, which was less than 0.05% over the whole FOV, as shown in Figure 11c.

## 4. Experimental Prototype and Captured Images

**Figure 12:**
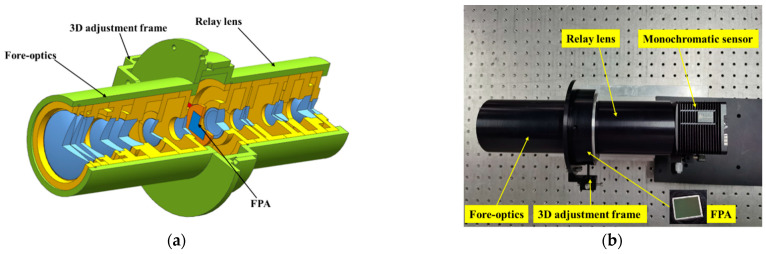
(**a**) Mechanical configuration of the system and (**b**) the experimental prototype on the test-bed.

In order to further verify the feasibility of our imaging scheme and optical design, we built an experimental prototype. We first developed the mechanical configuration of the system to check for possible problems in the fabrication and integration. Figure 12a shows a perspective view of this mechanical configuration of the system. Based on the mechanical model, we built the experimental prototype. As shown in Figure 12b, the experimental prototype consisted of a fore-optics, a PFA, a relay lens, and a monochromatic sensor. Firstly, we tested the back-working distance of the fore-optics and the object distance of the relay lens we built. Based on the test results, we then assembled the two system together with a spacer between them. By replacing the spacers with different thicknesses, we adjusted the image plane of the fore-optics and the object plane of the relay lens to the same position, according to the pattern captured by the monochromatic sensor. Then, we placed the PFA on a three-dimensional adjustment frame, which was fixed on the lens tube, and placed it between the fore-optics and relay lens. In the stage of coarse alignment, a rough position of the PFA with respect to the fore-optics and the relay lens was determined according to the images captured by the monochromatic sensor.

In our system, each voxel in the datacube was associated with a unique pixel on the monochromatic sensor. So, the center wavelength of each pixel needed to be determined, i.e., spectral calibration needed to be implemented before constructing the spectral images. As the center wavelength of each pixel was determined by the corresponding filter on the PFA, the spectral calibration was actually done to ensure the one-to-one correspondence between them.

**Figure 13:**
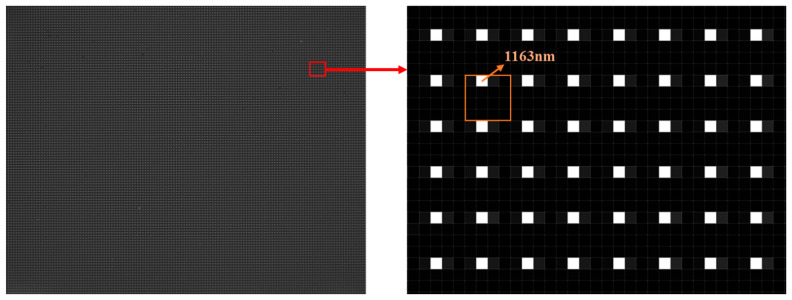
A raw image of monochromatic light with a wavelength of 1163 nm and its enlarged version.

For the spectral calibration, we illuminated an integrating sphere with a monochromator, and configured our imaging spectrometer to view the integrating sphere’s exit port. The wavelength of the light from the monochromator was then adjusted to the center wavelengths of filters on the PFA (1131 nm, 1163 nm, 1199 nm, 1238 nm, 1259 nm, 1301 nm, 1339 nm, 1381 nm, 1413 nm, 1456 nm, 1495 nm, 1532 nm, 1562 nm, 1600 nm, 1636 nm, and 1669 nm) successively. After passing through the fore-optics and PFA, the monochromatic light incidents upon the imaging sensor through the relay lens. The monochromatic sensor eventually converted light energy into an electrical signal and formed a digital image, which was displayed on a computer to provide live feedback. By observing the image pattern on a computer, the optimized alignment of the PFA both in rotation and in the X–Y–Z dimension were accomplished by operating the three-dimensional adjustment frame. The optimized alignment ensured that each filter corresponded to a unique pixel and the relationship between them was an optical conjugation of the relay lens. Then, the center wavelength of each pixel was obtained. A view of a raw image of 1163 nm from the monochromator experiment is presented in Figure 13, illustrating the response of the system to monochromatic light. As expected, only pixels that corresponded to the filters with a central wavelength of $\lambda_{2}=1163\mathrm{nm}$ light up, leaving the other pixels at a rather low intensity.

**Figure 14:**
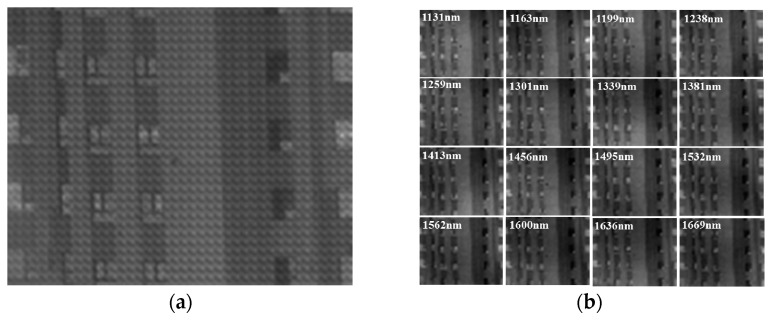
(**a**) Raw master image and (**b**) spectral images of the corresponding filters extracted from the raw master image.

Finally, we demonstrated the spectral imaging capability by taking a field experiment with the experimental prototype. Figure 14 shows a portion of imaging results of a building that was captured on a cloudy day. Figure 14a shows the raw master image. With the image decoding process, spectral images of the corresponding filters were continuously extracted from the raw master image, as shown in Figure 14b. As can be seen, the spatial resolution was good enough to recognize the windows and balconies in the scene.

In order to verify the imaging efficiency, the imaging times of the four images captured by our system were calculated. The imaging time was the sum of the exposure time and reconstruction time. Table 2 shows the calculation results of each image and their average values. From the table we can see, benefiting from the large relative aperture optical system and direct imaging mode, the imaging time was less than 0.14 s, which shows an excellent imaging efficiency. Compared with CASSI and ORRIS, the most representative computational and direct imaging spectrometer with imaging times of usually 1000 s and 4 s, respectively [32], our system possessed a better performance. Such a short imaging time enabled it to display the spectral images in real time and ensured its applications in time-crucial projects.

## 5. Discussion and Conclusions

In summary, we have presented a snapshot imaging spectrometer based on the pixel-level filter array (PFA). We designed the optical components of the system, including the fore-optics and relay lens, according to the parameters of the PFA selected. An experimental prototype was built based on our design. The prototype can capture 16 spectral channels over a spectral region of 900–1700 nm with a spatial resolution 160 × 128 pixels in a single integration time. Compared with the scanning imaging spectrometers, our system can acquire the 3D datacube within a single exposure, so it can be used for measuring dynamic scenes. In addition, it does not need additional equipment used for scanning imaging such as a turntable, thus reducing the complexity of the system. Another advantage of this method is that the stripe noise caused by image mosaicking of scanning system can be avoided. Compared with most of the existing snapshot imaging spectrometers, our system employs no dispersive or interferometric elements to separate the spectra by taking advantage of PFA, permitting the qualities of a compactness and low cost. Moreover, thanks to the large relative aperture optical system and simple cube assembly process, it needs less exposure time and computation load.

The snapshot imaging spectrometer based on the PFA can be used in many fields, such as autonomous driving and machine vision, which often need the capabilities of rapid spectral data acquisition and processing. In addition, it can be applied to medical diagnosis by simply adapting it to the commercial microscopies, such as confocal microscopy, fluorescence microscopy, electric endoscope, and fundus camera. Other potential applications include agricultural sensing, remote sensing, and food inspection.

Our system still has some limitations. First, it involves a tradeoff between spectral and spatial resolution, which prevents its applications in fields where the high spatial and spectral resolutions take precedence over the presence of temporal information. Second, as the number of channels in our system is determined by the PFA, it may have limitations in more accurate applications. Further works will focus on two aspects. First, we will try to solve the problem of spatial-spectral resolution tradeoff. Our system could be improved for acquiring high-resolution panchromatic images. Then, we can apply the pan-sharpening technique [33] to enhance the spatial resolution of the spectral images. Second, we would increase the number of channels by adding a filter wheel to the system.

## Figures and Tables

**Table 1:** Optical specifications of the fore-optics.

Parameters	Values
F-Number	4
Focal length	170 mm
FOV	4.2°
Waveband	900–1700 nm

**Table 2:** Imaging time of various images.

	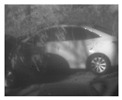	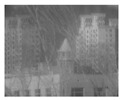	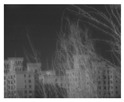	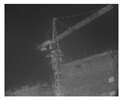	**Average Time**
Imaging time (s)	0.132	0.126	0.122	0.127	0.127

## Data Availability

The data presented in this study are available on request from the corresponding author. The data are not publicly available due to technical secrets.
